# The Significant Impacts of Interleukin-8 Genotypes on the Risk of Colorectal Cancer in Taiwan

**DOI:** 10.3390/cancers15204921

**Published:** 2023-10-10

**Authors:** Chia-Wen Tsai, Wen-Shin Chang, Te-Cheng Yueh, Yun-Chi Wang, Yu-Ting Chin, Mei-Due Yang, Yi-Chih Hung, Mei-Chin Mong, Ya-Chen Yang, Jian Gu, Da-Tian Bau

**Affiliations:** 1 Graduate Institute of Biomedical Sciences, China Medical University, Taichung 404333, Taiwan; wenwen816@gmail.com (C.-W.T.); halittlemelon@hotmail.com (W.-S.C.); s1245624@gmail.com (Y.-C.W.); tim9711118888@gmail.com (Y.-T.C.); viennaspring0312@gmail.com (Y.-C.H.); 2 Department of Epidemiology, The University of Texas MD Anderson Cancer Center, Houston, TX 77030, USA; 3 Terry Fox Cancer Research Laboratory, Department of Medical Research, China Medical University Hospital, Taichung 404327, Taiwan; andy820391@gmail.com (T.-C.Y.); d5218@mail.cmuh.org.tw (M.-D.Y.); 4 Division of Colon and Rectal Surgery, Department of Surgery, Taichung Armed Forces General Hospital, Taichung 41152, Taiwan; 5 National Defense Medical Center, Taipei 11490, Taiwan; 6 Department of General Surgery, China Medical University Hospital, Taichung 404332, Taiwan; 7 Department of Food Nutrition and Health Biotechnology, Asia University, Taichung 41354, Taiwan; mmong@asia.edu.tw (M.-C.M.); yachenyang@asia.edu.tw (Y.-C.Y.); 8 Department of Bioinformatics and Medical Engineering, Asia University, Taichung 41354, Taiwan

**Keywords:** colorectal cancer, genotype, IL-8, phenotype, polymorphism, Taiwan

## Abstract

**Simple Summary:**

The objective of this study was to investigate the association between *IL-8* rs4073, rs2227306, rs2227543, and rs1126647 genotypes and the risk of colorectal cancer (CRC) in the Taiwanese population. Our findings reveal that the A allele and AA genotype of *IL-8* rs4073 are significantly associated with an increased susceptibility to CRC in Taiwan. This observation provides further evidence of the complex interplay between inflammation and carcinogenesis in CRC development. Furthermore, individuals with the AA genotype exhibit significantly higher levels of serum IL-8 expression. Combining *IL-8* rs4073 genotyping with the assessment of IL-8 levels may have potential benefits in terms of the precise risk evaluation and early detection of CRC among patients.

**Abstract:**

Interleukin-8 (IL-8), a pro-inflammatory cytokine, is upregulated in CRC and plays an important role in its development and progression. Genetic variants in the *IL-8* gene may impact the risk of CRC by modulating IL-8 levels. Our primary objective was to investigate the role of *IL-8* genotypes in the development of CRC. To accomplish this, we employed the polymerase chain reaction-restriction fragment length polymorphism (PCR-RFLP) method to analyze the genotypes of *IL-8* rs4017, rs2227306, rs2227543, and rs1126647 in 362 CRC patients and 362 controls. Additionally, we evaluated the interactions between these genotypes and factors such as age, gender, smoking, alcohol consumption, and body mass index (BMI) status in relation to the risk of CRC. Furthermore, we utilized quantitative reverse transcription-PCR to measure the serum IL-8. The results demonstrated a significant difference in the distribution of rs4017 genotypes between the control and case groups (*p* for trend = 0.0059). Logistic regression analysis revealed that individuals with variant AA genotype had a 1.92-fold higher CRC risk (95% confidence interval [CI] = 1.28–2.89, *p* = 0.0023). Moreover, carriers of the *IL-8* rs4017 AT + AA genotypes exhibited a significant association with CRC risk (odds ratio [OR] = 1.39, 95% CI = 1.02–1.91, *p* = 0.0460). Additionally, individuals with *IL-8* rs4017 AA genotype displayed significantly elevated serum IL-8 compared to those with TT genotype at a 1.73–fold level (*p* < 0.0001), indicating a correlation between genotype and phenotype. In conclusion, the genotypes of *IL-8* rs4017, along with their associated expression levels, can potentially serve as predictive markers for the risk of CRC.

## 1. Introduction

Colorectal cancer (CRC) is a prevalent malignancy worldwide, ranking third in terms of incidence and second in cancer-related mortality [1]. Over the past two decades, significant progress has been made in CRC prognosis due to a deeper understanding of the biological mechanisms underlying colorectal carcinogenesis and tumor progression, along with advancements in treatment options [2]. In Taiwan, CRC is a major health concern, exhibiting the highest incidence rate among all cancer types and ranking third in terms of mortality, following lung and liver cancer. Given that 15–20% of CRC cases have a familial cancer history [3,4], genetic factors are considered to play a critical role in CRC etiology. Despite the identification of numerous genetic biomarkers for CRC in recent years [5,6,7,8], there remains great interest in identifying additional genetic susceptibility factors and exploring the interactions between genetic factors and other risk factors. These biomarkers included those that played important roles in extracellular microenvironment regulation [5,7], oncogenic miRNAs [6], DNA methylation homeostasis [8], etc. An enhanced understanding of the genetic contributions to CRC can assist scientists in developing more precise and targeted approaches to cancer prevention and therapy.

Chemokines play significant roles in CRC development and progression [9]. IL-8, encoded by the CXCL8 (chemokine C-X-C motif ligand 8) gene, stands out as a pro-angiogenic and pro-inflammatory chemokine. Previous studies have reported an increased expression of IL-8 mRNA in inflammatory colorectal polyps and advanced CRC tissues [10,11,12,13]. Through its interaction with receptors on target cells, IL-8 triggers specific downstream signaling pathways, including the phosphoinositide 3-kinase [PI3K] and mitogen-activated protein kinase [MAPK] cascades, consequently promoting various pro-tumoral phenotypes (reviewed in [10,11]). One of the well-established effects of tumor-derived IL-8 is VEGF-independent angiogenesis [11]. Additionally, IL-8 contributes to both the epithelial-to-mesenchymal transition (EMT) and the generation and maintenance of cancer stem cells [11]. Furthermore, immune cell populations also contribute to the production of IL-8, both within the tumor microenvironment (TME) and systemically [11]. Notably, IL-8 plays a pivotal role as a chemoattractant for monocytes/macrophages within the tumor tissue [11]. Moreover, IL-8 derived from tumor-associated macrophages enhances the metastatic behavior of CRC cells [10,11].

**Figure 1:**
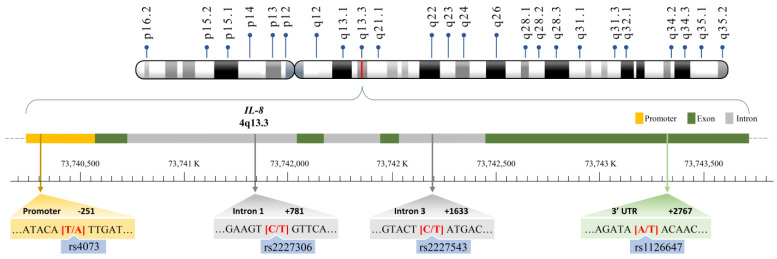
The physical map of the four *IL-8* polymorphic sites investigated in this study.

Single nucleotide polymorphisms (SNPs) are common subtle genetic variations that can impact the expression and/or function of specific genes, thereby contributing to tumorigenesis. While the biological role of IL-8 in cancer cell regulation and the tumor microenvironment has been well characterized, the significance of *IL-8* genotypes in CRC etiology remains unclear. Several SNPs within the *IL-8* gene, including T−251A (rs4073), C + 781T (rs2227306), C + 1633T (rs2227543), and A + 2767T (rs1126647), have been widely studied in their associations with different cancers (summarized in [14]); in particular, the A allele of *IL-8* rs4073, located in the promoter region of *IL-8*, is associated with the overexpression of the IL-8 protein [15,16,17]. Since IL-8 is a pro-angiogenic, pro-inflammatory, and pro-tumoral chemokine, it follows that this SNP may be predisposed to CRC. In this study, we aim to investigate the contribution of the rs4073, rs2227306, rs2227543, and rs1126647 genotypes of *IL-8* to the risk of CRC in Taiwan. This investigation may provide further evidence supporting the interplay between inflammation and carcinogenesis. Moreover, our objective is to provide a comprehensive summary that enables readers to gain a thorough understanding of the significance of *IL-8* genotypes in predicting the risk of CRC. The physical map for the SNPs investigated in this study is shown in Figure 1.

## 2. Materials and Methods

### 2.1. Study Population

The recruitment of CRC cases and healthy controls followed the methodology described in our previous publications [7,8]. In brief, 362 histologically confirmed CRC cases were recruited from the China Medical University Hospital (CMUH), and comprehensive pathological data were documented. Among them, 203 (56.1%) were males and 159 (43.9%) were females; a total of 95 (26.2%) were 60 years or younger, and 267 (73.8%) were older than 60 years. The stage distribution of the cases was as follows: 94 (26.0%) for stage 1, 72 (19.9%) for stage 2, 134 (37.0%) for stage 3, and 62 (17.1%) for stage 4. The inclusion criteria for the CRC case group included being over 30 years of age, having had their first CRC diagnosis within 6 months, and a willingness to participate in this study and donate blood samples. The inclusion criteria for the controls were as follows: not having a history of any malignancy, no diseases affecting dietary intake, no use of drugs that affected body weight, and a willingness to participate in this study. The same number of age- and gender-matched healthy subjects were chosen from the Health Examination Cohort database of the China Medical University Hospital. Some control participants were excluded and replaced with more properly recorded ones due to insufficient or incorrect data (*n* = 4) or a diagnosis of malignancy during the study (*n* = 8). All the participants are Taiwan citizens with a National Health Insurance card. The study protocol was approved by the Institutional Review Board of CMUH (approval code: DMR99-IRB-108).

### 2.2. Genotyping Methodology of IL-8 Polymorphisms

Genomic DNA was isolated from the blood samples using a Qiagen kit (Qiagen, Chatsworth, CA, USA). The genotyping of *IL-8* rs4073, rs2227306, rs2227543, and rs1126647 was performed using the polymerase chain reaction-restriction fragment length polymorphism (PCR-RFLP) method, as previously reported [18]. During the initial development of these assays, we sent ten DNA samples for DNA sequencing (AllBio, Taichung, Taiwan, ROC) with representative genotypes, and the results of the PCR-RFLP and sequencing were 100% concordant.

The PCR was performed in a PCR Thermocycler (Bio-RAD, Hercules, CA, USA) under the following conditions: initial denaturation at 94 °C for 5 min, followed by denaturation at 94 °C for 30 s, annealing at 64 °C for 40 s, and extension at 72 °C for 45 s. After 35 PCR cycles, a final extension step was performed at 72 °C for 10 min. The sequences of forward and reverse primers for *IL-8* rs4073, rs2227306, rs2227543, and rs1126647 are summarized in Table 1. The PCR products for rs4073, rs2227306, rs2227543, and rs1126647 were visualized using 3% agarose gel electrophoresis to confirm its successful amplification. Subsequently, the PCR products were digested with *Mfe* I, *EcoR* I, *Nla* III, and *BstZ*17 I, and the resulting digestion fragments were further confirmed by 4% agarose gel electrophoresis.

### 2.3. Quantitative Reverse Transcription Polymerase Chain Reaction for Examining IL-8 Transcriptional Expression

To assess the relationship between IL-8 mRNA expression and IL-8 SNPs, a total of 34 samples obtained from healthy controls with different genotypes were used. Total RNA was extracted from these samples using Trizol Reagent (Invitrogen, Carlsbad, CA, USA). The quantity of the total RNA was measured using a real-time quantitative RT-PCR instrument (FTC-3000, Funglyn Biotech Inc., Richmond Hill, ON, Canada). Glyceraldehyde 3-phosphate dehydrogenase (GAPDH) was employed as an internal quantitative control. For the amplification of IL-8 and GAPDH, the forward and reverse primers are summarized in Table 1 [18]. Fold changes were normalized based on the expression levels of GAPDH, and each assay was performed at least in triplicate.

### 2.4. Statistical Analysis

To compare the ages (a continuous variable) between the case and control groups, we employed the unpaired Student’s *t*-test. The distributions of gender, personal habits, different genotypes, and alleles among the subgroups were assessed using Pearson’s chi-square test. The associations between different genotypes and the risk of CRC were evaluated by calculating individual odds ratios (ORs) along with their corresponding 95% confidence intervals (CIs). Additionally, the expression levels among different genotypes were compared using the unpaired Student’s *t*-test. All statistical analyses were performed using the SPSS software version 12. Statistical significance was defined as a *p*-value less than 0.05.

## 3. Results

### 3.1. Characteristics of Study Population

The demographic and clinical characteristics of the cases and controls are presented in Table 2. The controls were matched 1:1 to the cases in terms of age and gender. There were no significant differences in the distribution of smoking frequency (*p* = 0.543), alcohol consumption (*p* = 0.441), and BMI (*p* = 0.181) between the case and control groups (Table 2).

### 3.2. Interluekin-8 Rs4073 Genotypes Were Specifically Associated with CRC Risk in Taiwan

The genotypes of *IL-8* rs4073, rs2227306, rs2227543, and rs1126647 in the control groups were consistent with the expected frequencies based on the Hardy–Weinberg equation (all *p* > 0.05). A significant association was observed between rs4073 genotypes and the risk of CRC. Compared to the wild-type TT genotype, individuals carrying the heterozygous variant genotype AT had an odds ratio (OR) of 1.20 (95% confidence interval [CI] = 0.85–1.68, *p* = 0.3407), while those carrying the homozygous variant AA genotype had a 1.92-fold increased risk of CRC (95% CI = 1.28–2.89, *p* = 0.0023) (*p* for trend = 0.0059). Individuals carrying the variant genotypes (AT + AA) had a 1.39-fold increased risk of CRC (95% CI = 1.02–1.91, *p* = 0.0460) (Table 3). However, no significant associations were found for rs2227306, rs2227543, or rs1126647 genotypes with the risk of CRC in any of the analyzed models (Table 3).

### 3.3. Validation of the IL-8 Alleles with CRC Risk

Table 4 presents the allelic test results for rs4073, rs2227306, rs2227543, and rs1126647 polymorphic sites in relation to CRC risk. Consistent with the findings in Table 3, the frequency of the A allele in rs4073 was significantly higher in the CRC patient group (49.4%) than the control group (41.2%). Individuals carrying the variant A allele had a 1.4-fold (95% CI = 1.14–1.72, *p* = 0.0018) increased risk of CRC. However, for rs2227306, rs2227543, and rs1126647, the frequencies of the variant alleles did not show significant differences between the cases and controls (Table 4).

### 3.4. Stratified Analyses of Interluekin-8 Rs4073 Genotypes by Age, Gender, Smoking, Alcohol Drinking, and BMI Status

We conducted stratified analyses to examine the association between *IL-8* rs4073 genotype and the risk of CRC based on age, gender, smoking, alcohol drinking, and BMI status (Table 5). In general, significant associations between the *IL-8* rs4073 genotype and CRC risk were observed in all the strata, except for the younger, smoker, and drinker subgroups. In the younger (OR = 1.78, 95% CI = 0.80–3.93, *p* = 0.2197), smoker (OR = 1.69, 95% CI = 0.72–3.95, *p* = 0.3195), and drinker (OR = 3.00, 95% CI = 0.79–11.46, *p* = 0.1862) subgroups, the risk associated with the AA genotype did not reach statistical significance, possibly due to the limited sample size (Table 5).

### 3.5. Genotype–Phenotype Correlation of IL-8 among Controls

**Figure 2:**
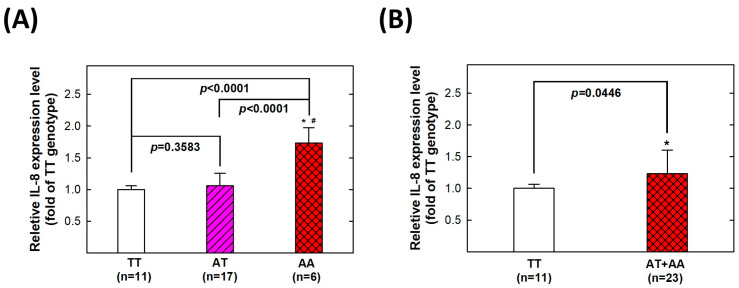
The genotype–phenotype correlation of *IL-8* rs4073. Correlation between the *IL-8* rs4073 genotype and IL-8 expression in the serum of healthy subjects. (**A**) comparing three different genotypes; (**B**) comparing TT and AT + AA genotypes. * Statistically significantly different from TT genotypes; # Statistically significantly different from AT genotypes. TT: TT genotype carriers; AT: AA genotype carriers; AA: AA genotype carriers.

We conducted further investigations on the serum expression levels of IL-8 and their correlation with *IL-8* rs4073 genotypes. A total of thirty-four serum samples from the control group were collected. Among these samples, 11 individuals had the TT genotype, 17 had the AT genotype, and 6 had the AA genotype at *IL-8* rs4073. We observed a significant increase of 1.73-fold in serum IL-8 levels in individuals with the homozygous variant genotype AA compared to those with the wild-type TT genotype (*p* < 0.0001) (Figure 2A). Furthermore, when combining the AT and AA genotypes, the expression of IL-8 remained significantly higher compared to the wild-type TT genotype (*p* = 0.0446) (Figure 2B).

## 4. Discussion

In this study, we found that the variant genotypes and allele of *IL-8* rs4073 were significantly associated with increased risks of CRC in Taiwan (Table 3 and Table 4). The other three SNPs—rs2227306, rs2227543, and rs1126647—were not associated with CRC risks in Taiwan. Furthermore, we found that the *IL-8* rs4073 AA genotype is associated with a higher IL-8 expression compared to the wild-type TT genotype (Figure 2). Our findings are consistent with several previous studies that reported an association between the *IL-8* rs4073 AA genotype and increased CRC risk in Caucasian populations [19,20,21] (Table 6). However, there were also conflicting results suggesting that the rs4073 AA genotype was not associated with CRC risks [22,23,24,25,26,27,28] (Table 6). The inconsistencies among these findings, despite studying comparable Caucasian populations, cannot be solely attributed to ethnic heterogeneity but may be influenced by factors such as small sample size, sampling bias, and other considerations. For instance, it is worth noting that the rs4073 genotype frequencies in studies conducted by Walczak et al. [19], Mustapha et al. [20], and Kury et al. [24] do not conform to the Hardy–Weinberg Equilibrium. Our study is the first and only one to report that the *IL-8* rs4073 AA genotype can serve as a marker for CRC risk in an Asian population. Further multi-population and multi-center studies encompassing larger sample sizes and diverse ethnicities are imperative for enhancing our understanding of the role of *IL-8* genotypes in the risk of CRC.

The *IL-8* rs4073 genotype, located in its promoter region, may play a crucial role in determining its expression levels in circulation, which has important clinical implications for CRC. For example, Burz et al. reported that CRC patients exhibit higher levels of IL-8 compared to healthy individuals, and elevated IL-8 levels are prognostic, and predictive factors for chemotherapy [29]. Moreover, high serum IL-8 levels have been associated with the expression of specific CD4+ T cell genes in CRC patients [30]. Furthermore, Oladipo et al. demonstrated that 65.4% of CRC tumor tissues expressed IL-8 within the tumor cores, while none of the normal colorectal tissues showed detectable IL-8 expression in inflammatory cells [31]. Two meta-analyses both concluded that high IL-8 levels significantly correlated with advanced CRC stages and increased mortality risk [11,32]. Conducting investigations that specifically measure IL-8 expression among CRC patients at different stages could have significant implications for understanding tumor progression and guiding the development of specific therapeutic strategies targeting the IL-8 axis. Additionally, the routine assessment of circulating IL-8 levels could be implemented to stratify CRC patients based on different prognoses and aid in selecting the most suitable treatment approach.

In retrospective epidemiological studies, the regions where the participants are selected from may confound the association between a potential risk factor and a disease. However, our study is genetics focused. Taiwan is a relatively small country, and the genetic background of our population is fully or nearly homogeneous. In addition, the study participants were all recruited from the China Medical University Hospital, the largest medical center in central Taiwan. Taiwan has an extremely high density of hospitals, and most citizens are accustomed to seeking medical care at facilities close to them. Nearly all of our participants were from central Taiwan, mostly residing in the city of Taichung and nearby areas. The effect of different regions on the genetic susceptibility of the same ethnicity is minimal.

## 5. Conclusions

In summary, this study provides evidence that the A allele and AA genotype of *IL-8* rs4073 are associated with an elevated risk of CRC in the Taiwanese population. The involvement of IL-8 and CRC is another piece of evidence supporting the intricate interplay between inflammation and carcinogenesis. Additionally, the AA genotype is linked to significantly higher levels of serum IL-8 expression among the control subjects. The variance of *IL-8* genotypes on its expression among CRC cases needs further investigation. In combination with *IL-8* rs4073 genotyping, increased IL-8 levels may benefit CRC patients by enabling more precise risk assessment and an early detection of the disease.

## Figures and Tables

**Table 1:** The sequences of primer pairs for genotyping and RT-PCR.

Polymorphic Sites	Primers
Genotyping	
rs4073	F: 5′-TCATCCATGATCTTGTTCTA-3′ R: 5′-GGAAAACGCTGTAGGTCAGA-3′
rs2227306	F: 5′-CTCTAACTCTTTATATAGGA-3′ R: 5′-GATTGATTTTATCAACAGGC-3′
rs2227543	F: 5′-CTGATGGAAGAGAGCTCTGT-3′ R: 5′-TGTTAGAAATGCTCTATATT-3′
rs1126647	F: 5’-CCAGTTAAATTTTCATTTCA-3’ R: 5’-CAACCAGCAAGAAATTACTA-3’
RT-PCR	
IL-8	F: 5′-AAACCACCGGAAGGAACCAT-3′ R: 5′-GCCAGCTTGGAAGTCATGT-3′
GAPDH	F: 5′-GAAATCCCATCACCATCTTCCAGG-3′ R: 5′-GAGCCCCAGCCTTCTCCATG-3′

F: Forward; R: reverse; RT-PCR: reverse transcription-polymerase chain reaction.

**Table 2:** Selected characteristics of the 362 CRC patients and 362 non-cancer controls.

Characteristic	Controls, *n* = 362		Cases, *n* = 362		*p*-Value ^a^
	*n*	%	*n*	%	
Age (years)					
≤60	95	26.2%	95	26.2%	1.0000
>60	267	73.8%	267	73.8%	
Gender					
Male	203	56.1%	203	56.1%	1.0000
Female	159	43.6%	159	43.9%	
Smoking					
Yes	84	23.2%	91	25.1%	0.5434
No	278	76.8%	271	74.9%	
Alcohol drinking					
Yes	51	14.1%	44	12.2%	0.4410
No	311	85.9%	318	87.8%	
BMI					
<24	175	48.3%	193	53.3%	0.1809
≥24	187	51.7%	169	46.7%	
Tumor size (cm)					
<5			195	53.9%	
≥5			167	46.1%	
Location					
Colon			257	71.0%	
Rectum			105	29.0%	
Lymph node involvement					
Negative			210	58.0%	
Positive			152	42.0%	
Stage					
1			94	26.0%	
2			72	19.9%	
3			134	37.0%	
4			62	17.1%	

SD, Standard deviation; BMI, body mass index; ^a^ based on the Chi-square test with Yates’ correction.

**Table 3:** Associations between *IL-8* genotypes and the risk of CRC in Taiwan.

SNP	Genotype	Cases	Controls	*p*-Value	OR (95% CI)
rs4073	TT	102 (28.2%)	128 (35.3%)		1.00 (Ref)
	AT	162 (44.7%)	170 (47.0%)	0.3407	1.20 (0.85–1.68)
	AA	98 (27.1%)	64 (17.7%)	0.0023 *	**1.92 (1.28–2.89)**
*P*_trend_				0.0059 *	
	AT + AA	260 (71.8%)	234 (64.7%)	0.0460 *	**1.39 (1.02–1.91)**
rs2227306	CC	144 (39.8%)	131 (36.2%)		1.00 (Ref)
	CT	162 (44.8%)	165 (45.6%)	0.5431	0.89 (0.65–1.23)
	TT	56 (15.4%)	66 (18.2%)	0.2804	0.77 (0.50–1.18)
*P*_trend_				0.4815	
	CT + TT	218 (60.2%)	231 (63.8%)	0.3582	0.86 (0.64–1.16)
rs2227543	CC	113 (31.2%)	122 (33.7%)		1.00 (Ref)
	CT	165 (45.6%)	163 (45.0%)	0.6643	1.09 (0.78–1.53)
	TT	84 (23.2%)	77 (21.3%)	0.4858	1.18 (0.79–1.76)
*P*_trend_				0.7185	
	CT + TT	249 (68.8%)	240 (66.3%)	0.5254	1.12 (0.82–1.53)
rs1126647	AA	127 (35.1%)	122 (33.7%)		1.00 (Ref)
	AT	169 (46.7%)	171 (47.2%)	0.8198	0.95 (0.68–1.32)
	TT	66 (18.2%)	69 (19.1%)	0.7726	0.92 (0.60–1.40)
*P*_trend_				0.9145	
	AT + TT	235 (64.9%)	240 (66.3%)	0.7543	0.94 (0.69–1.28)

OR: Odds ratio; CI: confidence interval; *p*-Values were calculated using the Chi-square test with Yates’ correction; HWE: Hardy–Weinberg Equilibrium; *P*_trend_, *p*-Value for trend analysis; *: *p* < 0.05; the significant values are marked in bold.

**Table 4:** Associations of *IL-8* alleles with the risk of CRC.

Allele	Cases	Controls	*p*-Value	OR (95% CI)
rs4073				
T	366 (50.6%)	426 (58.8%)		1.00 (Ref)
A	358 (49.4%)	298 (41.2%)	0.0018 *	**1.40 (1.14–1.72)**
rs2227306				
C	427 (59.0%)	450 (62.2%)		1.00 (Ref)
T	297 (41.0%)	274 (37.8%)	0.2368	1.14 (0.93–1.41)
rs2227543				
C	407 (56.2%)	391 (54.0%)		1.00 (Ref)
T	317 (43.8%)	333 (46.0%)	0.4280	0.91 (0.74–1.13)
rs1126647				
A	415 (57.3%)	423 (58.4%)		1.00 (Ref)
T	309 (42.7%)	301 (41.6%)	0.7095	1.05 (0.85–1.29)

*p*-Value was calculated using the Chi-square test with Yates’ correction; *: *p* < 0.05; the significant values are marked in bold.

**Table 5:** Associations between *IL-8* rs4073 genotypes and the risk of CRC in stratified analyses.

Genotype	Controls	Cases	OR (95% CI) ^a^	aOR (95% CI) ^b^	*p*-Value
Age					
≤60 years old					
TT	32	26	1.00 (ref)	1.00 (ref)	
AT	45	43	1.17 (0.60–2.29)	1.20 (0.63–2.23)	0.7576
AA	18	26	1.78 (0.80–3.93)	1.87 (0.83–3.76)	0.2197
>60 years old					
TT	96	76	1.00 (ref)	1.00 (ref)	
AT	125	119	1.20 (0.81–1.80)	1.29 (0.78–1.94)	0.4105
AA	46	72	**1.98 (1.23–3.19)**	**2.06 (1.27–3.36)**	0.0070 *
Gender					
Males					
TT	69	55	1.00 (ref)	1.00 (ref)	
AT	96	91	1.19 (0.75–1.88)	1.14 (0.79–1.79)	0.5291
AA	38	57	**1.88 (1.09–3.24)**	**1.83 (1.14–3.08)**	0.0308 *
Females					
TT	59	47	1.00 (ref)	1.00 (ref)	
AT	74	71	1.20 (0.73–1.99)	1.16 (0.71–2.05)	0.5503
AA	26	41	**1.98 (1.06–3.69)**	**2.09 (1.11–3.58)**	0.0451 *
Smoking behaviors					
Non-smokers					
TT	94	71	1.00 (ref)	1.00 (ref)	
AT	133	122	1.21 (0.82–1.80)	1.26 (0.84–1.93)	0.3863
AA	51	78	**2.02 (1.27–3.24)**	**2.17 (1.32–2.98)**	0.0044 *
Smokers					
TT	34	31	1.00 (ref)	1.00 (ref)	
AT	37	40	1.19 (0.61–2.30)	1.24 (0.59–2.43)	0.7362
AA	13	20	1.69 (0.72–3.95)	1.76 (0.77–4.08)	0.3195
Alcohol drinking behaviors					
Non-drinkers					
TT	101	84	1.00 (ref)	1.00 (ref)	
AT	150	144	1.15 (0.80–1.67)	1.22 (0.84–1.96)	0.5037
AA	60	90	**1.80 (1.17–2.79)**	**1.94 (1.19–2.93)**	0.0108 *
Drinkers					
TT	27	18	1.00 (ref)	1.00 (ref)	
AT	20	18	1.35 (0.56–3.23)	1.39 (0.58–3.37)	0.6508
AA	4	8	3.00 (0.79–11.46)	3.34 (0.73–8.65)	0.1862
BMI					
<24					
TT	57	49	1.00 (ref)	1.00 (ref)	
AT	81	86	1.24 (0.76–2.01)	1.19 (0.71–2.04)	0.4686
AA	37	58	**1.82 (1.04–3.20)**	**1.78 (1.14–2.95)**	0.0498 *
≥24					
TT	71	53	1.00 (ref)	1.00 (ref)	
AT	89	76	1.14 (0.72–1.83)	1.22 (0.70–2.29)	0.6584
AA	27	40	**1.98 (1.08–3.63)**	**2.15 (1.24–3.88)**	0.0370 *

^a^, by multivariate logistic regression analysis; ^b^, by multivariate logistic regression analysis after the adjustments of confounding factors; CI, confidence interval; aOR, adjusted odds ratio. *: *p* < 0.05; the significant values are marked in bold.

**Table 6:** Literature reports of the associations between *IL-8* rs4073 genotypes and the risk of CRC.

First Author	Year	Ethnicity	TT, AT, AA Genotype # of the Controls	TT, AT, AA Genotype # of the Cases	Highlights of the Findings	Ref #
Tsai	2023	Taiwanese	128:170:64	102:162: 98	AA genotype contributed to increased risk	current
Walczak	2012	Caucasian	99:71:35	50:104:37	AA genotype contributed to increased risk	[19]
Mustapha	2012	Mixed	54: 189: 12	40:183:32	AA genotype contributed to increased risk	[20]
Gunter	2006	Caucasian	65:94:32	52:87:66	AA genotype contributed to increased risk	[21]
Tsilidis	2009	Caucasian	114:162:86	65:88:52	No variant genotypes contributed to altered risk	[22]
Cacev	2008	Caucasian	53:73:34	46:75:39	No variant genotypes contributed to altered risk	[23]
Kury	2008	Caucasian	375:516:230	307:511:205	No variant genotypes contributed to altered risk	[24]
Wilkening	2008	Caucasian	115:296:169	71:133:96	No variant genotypes contributed to altered risk	[25]
Vogel	2007	Caucasian	160:367:226	83:178:94	No variant genotypes contributed to altered risk	[26]
Theodoropoulos	2006	Caucasian	64:90:42	76:106:40	No variant genotypes contributed to altered risk	[27]
Landi	2003	Caucasian	117:167:68	83:170:55	No variant genotypes contributed to altered risk	[28]

## Data Availability

The genotyping results and clinical data supporting the findings of this study are available from the corresponding authors upon reasonable request via email at artbau2@gmail.com.
